# Biological activity of soil contaminated with cobalt, tin, and molybdenum

**DOI:** 10.1007/s10661-016-5399-8

**Published:** 2016-06-08

**Authors:** Magdalena Zaborowska, Jan Kucharski, Jadwiga Wyszkowska

**Affiliations:** Department of Microbiology, University of Warmia and Mazury in Olsztyn, Plac Łódzki 3, 10 - 727 Olsztyn, Poland

**Keywords:** Cobalt, Tin, Molybdenum, Soil enzymes, Microbiological activity

## Abstract

In this age of intensive industrialization and urbanization, mankind’s highest concern should be to analyze the effect of all metals accumulating in the environment, both those considered toxic and trace elements. With this aim in mind, a unique study was conducted to determine the potentially negative impact of Sn^2+^, Co^2+^, and Mo^5+^ in optimal and increased doses on soil biological properties. These metals were applied in the form of aqueous solutions of Sn^2+^ (SnCl_2_^.^2H_2_O), Co^2+^ (CoCl_2_ · 6H_2_O), and Mo^5+^ (MoCl_5_), each in the doses of 0, 25, 50, 100, 200, 400, and 800 mg kg^−1^ soil DM. The activity of dehydrogenases, urease, acid phosphatase, alkaline phosphatase, arylsulfatase, and catalase and the counts of twelve microorganism groups were determined on the 25th and 50th day of experiment duration. Moreover, to present the studied problem comprehensively, changes in the biochemical activity and yield of spring barley were shown using soil and plant resistance indices—RS. The study shows that Sn^2+^, Co^2+^, and Mo^5+^ disturb the state of soil homeostasis. Co^2+^ and Mo^5+^ proved the greatest soil biological activity inhibitors. The residence of these metals in soil, particularly Co^2+^, also generated a drastic decrease in the value of spring barley resistance. Only Sn^2+^ did not disrupt its yielding. The studied enzymes can be arranged as follows for their sensitivity to Sn^2+^, Co^2+^, Mo^5+^: Deh > Ure > Aryl > Pal > Pac > Cat. Dehydrogenases and urease may be reliable soil health indicators.

## Introduction

In recent years, the attention of researchers has been focused mainly on heavy metals considered most harmful to the environment. These include, among others, Cd, Hg, Cr, Pb, and Cu (Wyszkowska et al. 2013; Zaborowska et al. 2015a). However, researchers have also increasingly focused on the contamination of soils with trace elements (Islam et al. 2015). This has its justification in that a common feature of all metals, regardless of their negative impact, is the fact that none of them biodegrade; they are characterized by a long biological half-life and show the potential to accumulate inside living organisms (Behbahaninia et al. 2009; Madrid et al. 2008). The side effects of their accumulation in the soil environment are therefore a problem which should be taken up and brought up for discussion, particularly in this age of dramatic industrialization and urbanization to meet the growing requirements of the human population.

Co^2+^ belongs to a group of transition metals. It most often assumes the +2, +3, and less often a +1 oxidation state. Only the ^59^Co isotope, representing 100 % of the isotopic composition of natural Co^2+^, is stable. It is a component of the following minerals: erythrite [Co_3_(AsO_4_)_2_^.^8H_2_O], glaucodot [(Co, Fe)AsS], and skutterudite (CoAs_3_) (Albanese et al. 2015; Shedd 2013). In Europe, the Mediterranean Basin countries are characterized by a higher Co^2+^ content than Northern Europe. This phenomenon is closely related to ophiolitic rocks (mafic and ultramafic) (Albanese et al. 2015). Contamination of soils with Co^2+^ is mainly caused by mining and smelting activity, fertilizer use, and sewage sludge spreading (Hamilton 2000). The production of liquid catalysts used in refineries is a source of environmental pollution with both Co^2+^ and Mo^5+^ (Lewis et al. 2012). In soil, this metal is closely correlated with Mn, Fe, and Al (Sterckeman et al. 2006). The bioavailability of Co^2+^ and, thus, its toxicity is also affected by the physicochemical properties of the soil environment such as structure, organic matter, pH, and complexing compounds (Luo 2010). Exposure to increased amounts of Co^2+^ in soil causes side effects both among soil microorganisms (Lock et al. 2006) and plants (Chatterjee and Chatterjee 2003). The response to an excess of Co^2+^ in a plant is heightened activity of superoxide dismutase (SOD), an enzyme responsible for O_2_ dismutation and an increase in iron sequestration and ferritin synthesis (Tewari et al. 2002). Co^2+^ plays a quite important role in the human body because it is the central atom of cobalamin, a coenzyme precursor, whose deficiency causes anemia (Paustenbach et al. 2013). Nevertheless, its genotoxic properties were also discovered, which should be taken into account in the overall Co^2+^ assessment (Kirkland et al. 2015).

The average Sn^2+^ content of the Earth’s crust is estimated at 2.5 mg kg^−1^. This metal is a component of cassiterite (SnO_2_), stannite (Cu_2_FeSnS_4_), teallite (PbSnS_2_), and montesite (PbSn_4_S_5_) (Pendias and Pendias 2001). In 2012, the world Sn^2+^ production amounted to 230 Mg (metric tons). The main leaders were China, Indonesia, Peru, and Bolivia (USDI 2013). Because Sn^2+^ is a component of nuclear waste, including ^235^U fission products, where the half-life of ^126^Sn is 10^5^ years and of ^121^Sn 55.5 years (National Research Council 1983), the effects of soil Sn^2+^ mobility should be examined. Organotin compounds (OTC) which are part of fungicides, insecticides, bactericides, wood preservatives, and PVC stabilizers pose a potential threat to the environment (Hoch 2001). The inorganic tin forms are less toxic, but the effects of their soil presence are also less known (Marcic et al. 2006). Tendencies have been observed for the affinity of Sn^2+^ mobility to the soil size fraction and its organic matter content (Sterckeman et al. 2006). Due to its low soil solubility, Sn^2+^ translocation and uptake by plants is minimal, but increases with decreasing pH (Nakamaru and Uchida 2008).

Mo^5+^, as a trace element, is necessary in the environment in small amounts. The sources of excessive Mo^5+^ emission to the environment are mining; biosolids; fertilizers; the production of alloys, catalysts, and coal; and petroleum combustion (Mcgrath et al. 2010; Buekers et al. 2010). It occurs in the soil in the form of an oxyanion, in aluminosilicates and organic matter (Pyrzyńska 2007). In the soil environment, the Mo^5+^ amount is correlated with the presence of Fe, Al, and organic carbon content (Sterckemann et al. 2006), while with Cu^2+^, they are antagonists (Pyrzyńska 2007). In acidic soils, the dominant, soluble Mo^5+^ form is the anion MoO_4_^2−^. In neutral and alkaline medium, Mo^5+^ forms mobile anionic complex compounds (Buekers et al. 2010). The biological role of Mo^5+^ is based on co-forming the pterin complex (a Mo cofactor), binding with enzymes participating in nitrogen (nitrate reductase) and sulfur (sulfite oxidase) metabolism, purine catabolism, and hormone biosynthesis. Over 50 enzymes of bacterial origin containing Mo^5+^ have been identified (Mendel and Bittner 2006). Nevertheless, both a deficiency and excessive exposure to this metal can cause abnormalities in the functioning of living organisms and, thus, ecosystems (Mcgrath et al. 2010), although according to Das et al. (2007), its direct effect on the metabolic processes of microorganisms is relatively low. An increased content of this element can possibly reduce nitrogen fixation.

Soil condition is closely related to microorganism activity and is considered a reliable indicator of the impact of environmental stress on soil (Epelde et al. 2008). Since the effects and range caused by contamination of soils with Sn^2+^, Co^2+^, and Mo^5+^ have not yet been widely studied, determining the biological activity of a soil environment subjected to the pressure of this group of metals seems a necessary step in a strategy to assess the scale of the problem related to Sn^2+^, Co^2+^, and Mo^5+^ accumulation or question the justifiability of the formulated hypothesis.

## Materials and Methods

### Experimental. Soil sampling and samples preparation

The study object was soil material from the Didactic and Experimental Center in Tomaszkowo. The area designated for research purposes, including protective belts, occupies around 4.5 ha. Soil with a granulometric composition of loamy sand (fraction of sand is 75 %, silt—17 %, clay—8 %) determined according to the US Department of Agriculture’s particle size distribution classification was collected from the arable-humus horizon of typical brown soils (Eutric Cambisol). The studied soil is characterized by the following properties: pH w 1 mol KCl dm^3^—6.3, C_org_ kg^−1^ dm of soil—6.4, hydrolytic acidity (HAC)—8.4 mmol^(+)^ per kilogram of soil, sum of exchangeable bases (TEB) Ca^2+^, Mg^2+^, K^+^, and Na^+^—84 mmol^(+)^ per kilogram of soil, cation exchange capacity (CEC)—92.40 mmol^(+)^ per kilogram of soil, and base saturation (BS)—90.91 %. The next stage of the experiment was conducted in the greenhouse of the University of Warmia and Mazury in Olsztyn (NE Poland) based on a pot test, replicated five times.

The effect of the following variable factors was assessed: (1) the type of the heavy metals used: Sn^2+^ (SnCl_2_ · 2H_2_O), Co^2+^ (CoCl_2_ · 6H_2_O), and Mo^5+^ (MoCl_5_); (2) the degree of soil contamination with Sn^2+^, Co^2+^, and Mo^5+^ in milligrams per kilogram soil DM: 0, 25, 50, 100, 200, 400, and 800; and (3) test duration: 25 and 50 days. An analysis of the impact of the three metals on the yield of spring barley cv. *Rabel* was also performed.

Before conducting the experiment, the soil material was prepared by contaminating it with the individual heavy metals and adding NPKMg fertilizers. After mixing the soil in a polyethylene vessel and packing it in pots (3.5 dm^3^), in the amount of 3.2 kg per pot, the soil moisture in all the objects was brought to the level of 60 % of capillary water capacity. One level of fertilization with macro- and microelements was used and was expressed on an elemental basis in milligrams per kilogram soil: N—250 [CO(NH_2_)_2_], P—50 (KH_2_PO_4_), K—90 (KH_2_PO_4_), Mg—20 (MgSO_4_^.^ 7H_2_O), Cu—5 (CuSO_4_ · 5H_2_O), Zn—5 (ZnCl_2_), Mo—5 (NaMoO_4_ · 2H_2_O), Mn—5 (MnCl_2_ · 4H_2_O), and oraz B—0.33 (H_3_BO_3_).

The vegetation of spring barley cv. *Rabel* was conducted for 50 days. Fifteen plants were left in each pot after seedling. The plant dry matter yield was determined after spring barley harvest at stage (according to the BBCH scale) 52—heading (20 % of inflorescence emerged).

### Microbiological and biochemical analysis

In each soil sample, the activity of the enzymes dehydrogenases, catalase, urease, acid phosphatase, and alkaline phosphatase were determined in three replications, according to the methods given in Tables 1 and 2. Soil biochemical activity, except for catalase, was established using a Perkin-Elmer Lambda 25 spectrophotometer (MA, USA). The analyses of soil were performed after 25 and 50 days of the experiment.

**Table 1:** Determined soil enzymes

No.	Enzyme	Substrate	Unit	References
1	Dehydrogenases (EC 1.1)	2,3,5-triphenyl tetrazolium chloride	Triphenyl formazan (μmol kg^−1^ dm of soil h^−1^)	Öhlinger (1996)
2	Catalase (EC 1.11.1.6)	H_2_O_2_—aqueous solution	O_2_ (mol kg^−1^ dm of soil h^−1^)	Alef and Nannpieri (1998)
3	Urease (EC 3.5.1.5)	Urea—aqueous solution	N-NH_4_ (mmol kg^−1^ dm of soil h^−1^)	
4	Acid phosphatase (EC 3.1.3.2) alkaline phosphatase (EC 3.1.3.1)	Disodium—4-nitrophenylphosphate hexahydrate	p-nitrophenol (mmol kg^−1^ dm of soil h^−1^)	
5	Arylsulphatase (EC 3.1.6.1)	Potassium-4-nitrophenyl-sulfate

**Table 2:** Activity enzymes in soil contaminated of Sn^2+^, Co^2+^, and Mo^5+^ (for average values of the research time) (kg^1^ DM soil h^−1^)

Dose heavy metals (mg kg^−1^)	Deh	Ure	Pac	Pal	Cat	Aryl
	μmol TFF	mmol N-NH_4_	mmol PNP	mmol PNP	mol O_2_	mmol PNP
Sn^2+^						
0	9.254 ± 0.063	1.050 ± 0.020	2.245 ± 0.066	2.195 ± 0.017	0.275 ± 0.008	0.236 ± 0.008
25	8.572 ± 0.070	0.954 ± 0.024	2.116 ± 0.060	2.161 ± 0.009	0.274 ± 0.013	0.215 ± 0.000
50	8.442 ± 0.083	0.911 ± 0.016	2.088 ± 0.017	2.118 ± 0.017	0.273 ± 0.008	0.204 ± 0.000
100	8.011 ± 0.044	0.923 ± 0.024	2.032 ± 0.024	2.095 ± 0.026	0.271 ± 0.010	0.204 ± 0.000
200	7.405 ± 0.125	0.882 ± 0.026	2.008 ± 0.023	1.970 ± 0.017	0.266 ± 0.013	0.174 ± 0.000
400	6.549 ± 0.081	0.800 ± 0.022	2.042 ± 0.032	1.912 ± 0.017	0.259 ± 0.012	0.177 ± 0.000
800	5.579 ± 0.072	0.648 ± 0.038	1.991 ± 0.000	1.803 ± 0.009	0.249 ± 0.010	0.166 ± 0.000
Average	7.687	0.881	2.074	2.036	0.267	0.194
r	−0.938	−0.872	−0.584	−0.850	−0.854	−0.868
Co^2+^						
0	8.551 ± 0.128	1.040 ± 0.037	2.263 ± 0.087	2.202 ± 0.009	0.276 ± 0.007	0.235 ± 0.000
25	6.714 ± 0.158	0.911 ± 0.019	2.061 ± 0.079	1.964 ± 0.032	0.266 ± 0.011	0.207 ± 0.000
50	4.836 ± 0.078	0.832 ± 0.033	2.032 ± 0.023	1.755 ± 0.000	0.251 ± 0.011	0.191 ± 0.000
100	2.971 ± 0.167	0.765 ± 0.019	1.941 ± 0.036	1.769 ± 0.018	0.239 ± 0.007	0.183 ± 0.000
200	2.102 ± 0.042	0.628 ± 0.021	1.921 ± 0.067	1.573 ± 0.033	0.200 ± 0.009	0.169 ± 0.000
400	2.138 ± 0.014	0.649 ± 0.014	1.799 ± 0.027	1.476 ± 0.027	0.200 ± 0.009	0.168 ± 0.000
800	1.940 ± 0.023	0.309 ± 0.019	1.760 ± 0.018	1.370 ± 0.027	0.194 ± 0.011	0.155 ± 0.000
Average	4.179	0.733	1.968	1.730	0.232	0.187
r	−0.677	−0.902	−0.752	−0.791	−0.797	−0.735
Mo^5+^						
0	8.720 ± 0.187	1.016 ± 0.023	2.218 ± 0.065	2.208 ± 0.026	0.272 ± 0.008	0.252 ± 0.008
25	7.708 ± 0.059	0.885 ± 0.009	2.126 ± 0.025	2.189 ± 0.039	0.243 ± 0.009	0.239 ± 0.000
50	6.599 ± 0.034	0.640 ± 0.025	2.025 ± 0.040	2.090 ± 0.018	0.241 ± 0.008	0.206 ± 0.000
100	6.170 ± 0.082	0.654 ± 0.012	1.894 ± 0.024	2.078 ± 0.018	0.226 ± 0.012	0.170 ± 0.000
200	5.075 ± 0.054	0.605 ± 0.012	1.778 ± 0.015	2.004 ± 0.018	0.223 ± 0.013	0.167 ± 0.000
400	3.467 ± 0.084	0.527 ± 0.016	1.540 ± 0.039	1.707 ± 0.009	0.211 ± 0.009	0.141 ± 0.000
800	1.681 ± 0.081	0.152 ± 0.010	1.463 ± 0.033	1.386 ± 0.028	0.195 ± 0.010	0.087 ± 0.000
Average	5.631	0.640	1.863	1.952	0.230	0.180
r	−0.929	−0.888	−0.892	−0.988	−0.811	−0.915
LSD_0.05_	a—0.654 b—0.043 a, b—0.113	a—0.016 b—0.010 a, b—0.028	a—0.032 b—0.021 a, b—0.055	a—0.015 b—0.010 a, b—0.026	a—0.007 b—0.005 a, b—0.013	a—0.161 b—0.105 a, b—0.279

*a* dose of heavy metal, *b* kind of heavy metal, *Deh* dehydrogenases, *Ure* urease, *Pac* acid phosphatase, *Pal* alkaline phosphatase, *Cat* catalase, *Aryl* arylsuphatase

On days 25 and 50 of the experiment, the soil samples were tested for the number of twelve microorganism groups: cellulolytic bacteria, ammonification bacteria, nitrogen-immobilizing bacteria, *Arthrobacter* sp., *Azotobacter* sp., and *Pseudomonas* sp. on a medium described by Wyszkowska et. al. (2008), Actinobacteria—on the medium developed by Küster and Williams with the addition of nystatin and actidione (Parkinson et al. 1971), fungi—on Martin medium (1950), and copiotrophic bacteria, copiotrophic spore-forming bacteria, oligotrophic bacteria, and oligotrophic spore-forming bacteria on Onta and Hattori (1983) medium. The number of microorganisms was determined with a colony counter.

### Calculations and statistical analysis

The activity of soil enzymes and spring barley yield were used to determine soil and plants resistance (RS) to Sn^2+^, Co^2+^, and Mo^5+^ contamination. Calculations were made with a formula proposed by Orwin and Wardle (2004):

$$ \mathrm{R}\mathrm{S} = 1-\frac{2\ \left|\ \mathrm{D}0\left.\right|\right.}{\mathrm{C}0 + \left|\mathrm{D}0\left.\right|\right.} $$

where: D_0_—difference between control soil (C_0_) and contaminated soil after 25 days of incubation (t_0_). The values of RS remain in the range of 0 to 1, where 1 indicates strong soil resistance, i.e., negligible effects of external factors. The lower the resistance, the stronger the impact of a given factor on the soil environment.

The results were processed statistically using Statistica 10.0 software (StatSoft, Inc. 2012). Homogeneous groups were calculated by Tukey’s test, at *p* = 0.01. Pearson’s simple correlation coefficients between increasing Sn^2+^, Co^2+^, and Mo^5+^ doses and the activity of individual enzymes were also determined. The effect of the time of soil residence of the tested metals on its biochemical activity was illustrated using principal component analysis (PCA). The reaction of microorganisms to soil contamination with Sn^2+^, Co^2+^, and Mo^5+^ was analyzed using a clustering method (Ward’s method dendrogram). The percentage of variation for all analyzed variables (η^2^) was determined with the analysis of variance (ANOVA).

## Results and discussion

**Fig. 1:**
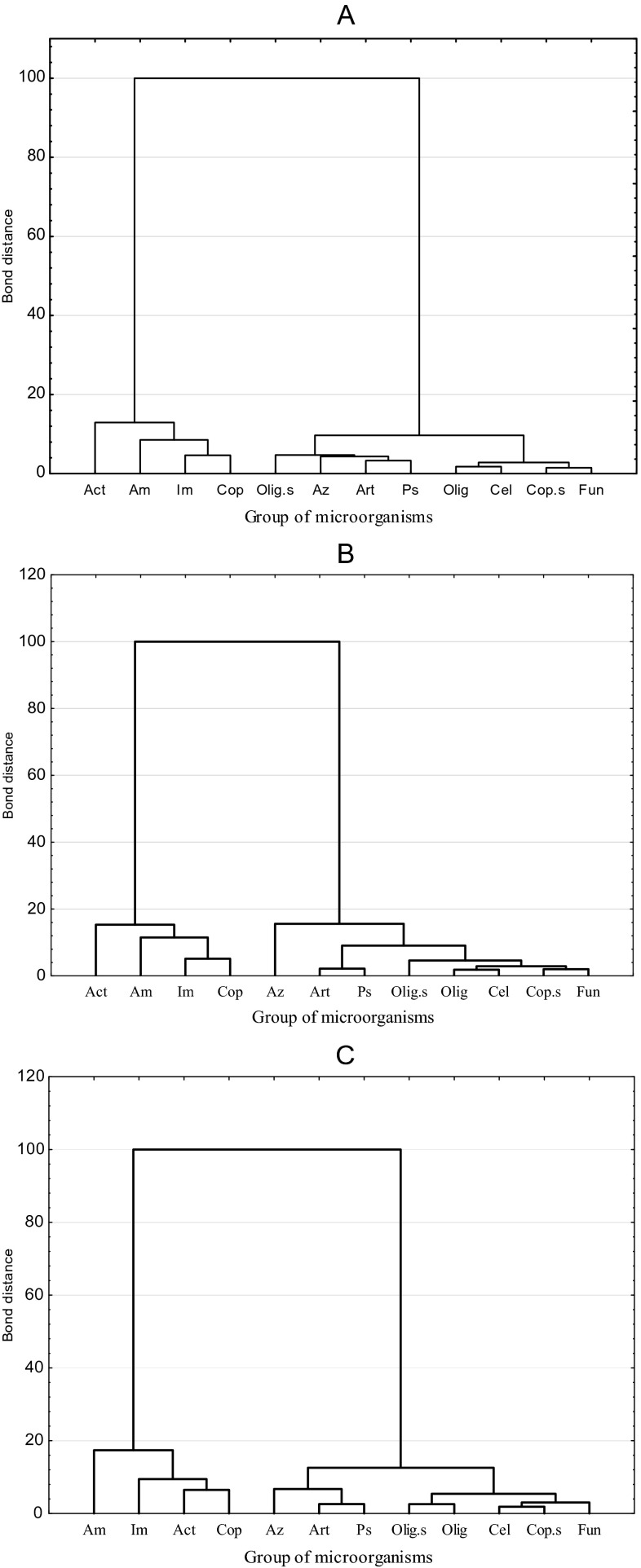
Similarity of microbial reaction to contamination of soil with **a** Sn^2+^, **b** Co^2+^, and **c** Mo^5+^. *Am* ammonifying bacteria, *Im* nitrogen-immobilizing bacteria, *Act* Actinobacteria, *Cop* copiotrophic bacteria, *Cop.s* spore-forming copiotrophic bacteria, *Az Azotobacter* sp., *Art. Arthrobacter* sp., *Ps. Pseudomonas* sp., *Olig* oligotrophic bacteria, *Olig.s* spore-forming oligotrophic bacteria, *Cel* cellulolytic bacteria, *Fun* fungi

A significant inhibitory impact of all the used metals on soil biological properties was noted in the conducted study. This thesis can be substantiated by high values of the η^2^ coefficient. The major factor modifying the condition of the soil subjected to the pressure of Sn^2+^, Co^2+^, and Mo^5+^ to the highest degree was the dose (54.25 %) and, to a lower degree, the xenobiotic type (11.7 %). The reaction of microorganisms to individual metals was similar (Fig. 1a–c). This was illustrated in cluster analysis diagrams by Ward’s method. Two separate clusters consisting of several subclusters with homogeneous variances were formed by the following: ammonifying, nitrogen-immobilizing, copiotrophic bacteria and Actinobacteria (the first cluster) and *Arthrobacter* sp., *Azotobacter* sp., *Pseudomonas* sp., oligotrophic bacteria, oligotrophic spore-forming bacteria, cellulolytic bacteria, fungi, and copiotrophic spore-forming bacteria (the second cluster). The obtained tendencies are puzzling because *Pseudomonas* sp., due to its ability to produce exopolysaccharide (EPS) responsible for biosorption or bioaccumulation, acquires features of resistance to heavy metals (Kilic and Donmez 2008). On the other hand, genus *Azotobacter* bacteria, which were located within the same cluster, are considered some of most sensitive to this group of xenobiotics (Borowik et al. 2014). Wang et al. (2010) suggest that gram-positive bacteria are more susceptible to contamination with heavy metals than gram-negative bacteria. In their toxicity ranking, Co^2+^ took the place: Cr > Pb > As > Co > Zn > Cd > Cu. Root exudates accumulating in the barley rhizosphere, including: glucose, glutamic acid, citric acid, and oxalic acid (Renella et al. 2006), which are not neutral for microbiological activity and diversity, could also prove a factor moderating the reactions of individual microorganism groups.

Based on an analysis of changes in soil biochemical properties, it can be clearly stated that regardless of the type of applied metal, dehydrogenases, and urease are the most sensitive enzymes (Table 1). In the objects with 800 mg Sn^2+^, Co^2+^, and Mo^5+^ per kilogram soil, their activity decreased, respectively, by: 39.71 and 38.28 %, 77.37 and 70.28 %, and 80.72 and 85.04 % compared to control samples. Taking into account the sensitivity of individual enzymes to the tested metals, they can be arranged as follows: for Sn^2+^, Deh > Ure > Aryl > Pal > Pac > Cat; for Co^2+^, Deh > Ure > Pal > Aryl > Cat > Pac; and for Mo^5+^, Ure > Deh > Aryl > Pac > Cat > Pal. Each metal showed varied toxicity. Based on own research, Co^2+^ can be considered the strongest inhibitor of biochemical properties. Under its pressure, enzymatic activity was inhibited, on average, by 25.24 %. Mo^5+^ ranked second (24.00 %) and Sn^2+^ third (11.42 %).

**Fig. 2:**
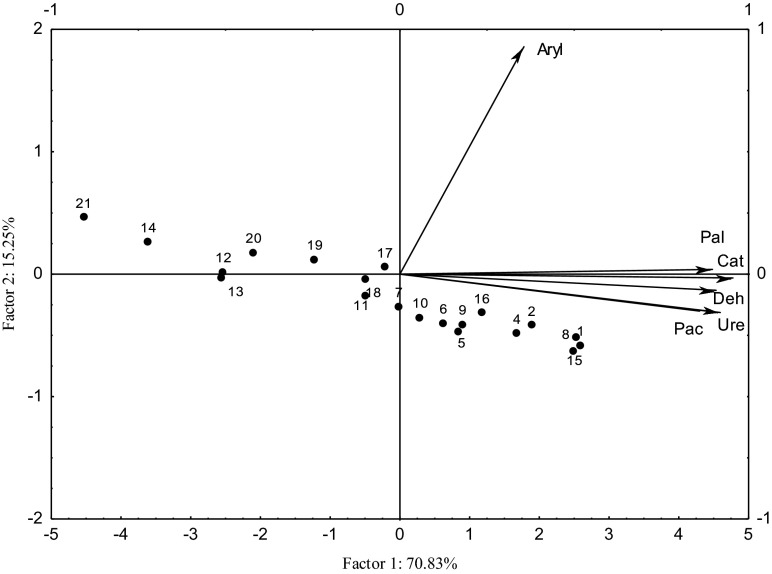
Enzyme activity in soil contaminated with Sn^2+^, Co^2+^, and Mo^5+^ after 25 days of experiments—PCA method. *Vectors* represent the analyzed variables. *Deh* dehydrogenases, *Ure* urease, *Pal* alkaline phosphatase, *Pac* acid phosphatase, *Cat* catalase. Dose Sn^2+^, Co^2+^, and Mo^5+^ mg kg^−1^ DM soil: 0 (cases 1, 8, 15), 25 (2, 9, 16), 50 (3, 10, 17), 100 (4, 11, 18), 200 (5, 12, 19), 400 (6, 13, 20), and 800 (7, 14, 21). Cases 1–7 with Sn^2+^, 8–14 with Co^2+^, and 15–21 with Mo^5+^

**Fig. 3:**
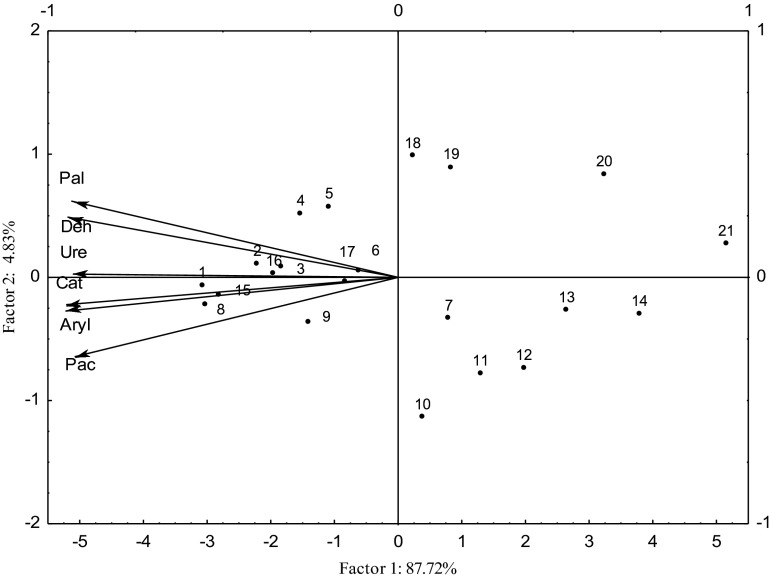
Enzyme activity in soil contaminated with Sn^2+^, Co^2+^, Mo^5+^ after 50 days of experiments—PCA method. *Vectors* represent the analyzed variables. *Deh* dehydrogenases, *Ure* urease, *Pal* alkaline phosphatase, *Pac* acid phosphatase, *Cat* catalase. Dose Sn^2+^, Co^2+^, and Mo^5+^ mg kg^−1^ DM soil: 0 (cases 1, 8, 15), 25 (2, 9, 16), 50 (3, 10, 17), 100 (4, 11, 18), 200 (5, 12, 19), 400 (6, 13, 20), and 800 (7, 14, 21). Cases 1–7 with Sn^2+^, 8–14 with Co^2+^, and 15–21 with Mo^5+^

A PCA analysis including the time of soil Sn^2+^, Co^2+^, and Mo^5+^ residence revealed detailed relationships important in the study (Figs. 2 and 3). Both after 25 and 50 days of the experiment, the distribution of vectors around the axis representing the first principal component describing 70.83 and 87.72 % of the total data variance, respectively, indicates that regardless of heavy metal type, the activity of all the enzymes was positively correlated with this variable. Arylsulfatase activity was of the greatest importance only in the objects incubated for 25 days, for the second principal component defining 15.25 %. The distances between cases and the values of their coordinates indicate a negative effect of both Co^2+^ and Mo^5+^ in doses above 200 mg metal per kilogram soil DM (Fig. 2). After 50 days of study duration, a higher toxic impact of Co^2+^ was manifested—from the dose of 100 mg Co^2+^ per kilogram soil DM, and for Mo^5+^ after the application of 400 and 800 mg Mo^5+^ per kilogram soil DM (Fig. 3). Soil contamination with Sn^2+^ did not significantly disturb its biochemical properties in either of these objects.

The problem of soil stability, besides microbiological and biochemical indicators, defines its quality in a broader spectrum (Griffiths and Phillipot 2013). Therefore, the soil resistance index (RS) dealing with this phenomenon was calculated in the study (Table 3). After the application of 25 mg Sn^2+^, Co^2+^, and Mo^5+^ per kilogram soil DM, the lowest resistance values were noted in the case of Sn^2+^ and Mo^5+^ for urease and Co^2+^ for dehydrogenases. The RS index also highlighted the interrelationship between deepening soil homeostasis disturbance and increasing the inhibition power of the tested heavy metals. The soil balance was disturbed most severely by Co^2+^, followed by Mo^5+^ and Sn^2+^, reducing the resistance of all the enzymes, on average, by 32.35, 30.48, and 11.47 % compared to the samples with the lowest dose of the metals.

**Table 3:** Indicators of enzymes resistance (RS) to soil contamination with Sn^2+^, Co^2+^, and Mo^5+^ after 50 days of the research

Dose heavy metals (mg kg^−1^)	Deh	Ure	Pac	Pal	Cat	Aryl
Sn^2+^						
25	0.863^ab^	0.763^a^	0.905^abc^	0.952^ab^	0.958^ab^	0.772^b^
50	0.823^b^	0.752^a^	0.860^abcd^	0.853^de^	0.992^a^	0.780^b^
100	0.811^b^	0.742^a^	0.756^cde^	0.865^cd^	0.992^a^	0.784^b^
200	0.717^c^	0.762^a^	0.792^abcd^	0.874^cd^	0.877^bc^	0.631^c^
400	0.511^f^	0.758^a^	0.798^abcd^	0.770^f^	0.832^c^	0.651^c^
800	0.406^g^	0.355^e^	0.783^bcd^	0.624^g^	0.827^c^	0.503^d^
average	0.688	0.689	0.816	0.823	0.913	0.687
r	−0.964^*^	−0.883^*^	−0.478	−0.959^*^	−0.833^*^	−0.933^*^
Co^2+^						
25	0.654^d^	0.753^a^	0.888^abc^	0.800^f^	0.915^abc^	0.781^b^
50	0.353^g^	0.500^d^	0.850^abcd^	0.556^h^	0.840^c^	0.610^c^
100	0.193^h^	0.536^cd^	0.774^bcd^	0.576^gh^	0.706^d^	0.507^d^
200	0.114^i^	0.526^cd^	0.743^cde^	0.552^h^	0.593^ef^	0.496^d^
400	0.111^i^	0.469^d^	0.593^ef^	0.499^i^	0.601^ef^	0.491^d^
800	0.111^i^	0.191^f^	0.588^ef^	0.435^j^	0.554^f^	0.385^e^
average	0.256	0.496	0.739	0.570	0.702	0.545
r	−0.618	−0.884^*^	−0.895^*^	−0.727^*^	−0.781^*^	−0.775^*^
Mo^5+^						
25	0.893^a^	0.659^ab^	0.961^a^	0.989^a^	0.838^c^	0.920^a^
50	0.597^de^	0.565^bcd^	0.937^ab^	0.924^b^	0.833^c^	0.619^c^
100	0.591^e^	0.617^bc^	0.760^cde^	0.913b^c^	0.680^de^	0.504^d^
200	0.487^f^	0.570^bcd^	0.705^def^	0.813^ef^	0.660^de^	0.484^d^
400	0.202^h^	0.323^e^	0.543^f^	0.589^gh^	0.556^f^	0.370^e^
800	0.150^hi^	0.032^g^	0.548^f^	0.394^j^	0.534^f^	0.199^f^
average	0.486	0.461	0.742	0.770	0.683	0.516
r	−0.870^*^	−0.984^*^	−0.839^*^	−0.983^*^	−0.849^*^	−0.842^*^

The same superscripted letters in the columns indicate homogeneous groups; n = 17

*r* correlation coefficient, *Deh* dehydrogenases, *Ure* urease, *Pac* acid phosphatase, *Pal* alkaline phosphatase, *Cat* catalase, *Aryl* arylsuphatase

^*^significant for *p* = 0.01

Many researchers share the view that a decrease in the enzymatic activity of soils is a manifestation of abiotic stress caused by accumulated heavy metals in excessive amounts (Kucharski et al. 2011; Wyszkowska et al. 2013; Zaborowska et al. 2015b; Xian et al. 2015). Dehydrogenases are considered the most sensitive parameters used to assess the effects of soil environment contamination (Gil-Sotres et al. 2005). In the toxicity series (ED_50_) for dehydrogenases, Co^2+^ was placed as follows: Hg (2 mg) > Cu (35 mg) > Cr^6+^ (71 mg) > Cr^3+^ (75 mg) > Cd^2+^ (90 mg) > Ni^2+^ (100 mg) > Zn^2+^ (115 mg) > As^3+^ (168 mg) > Co^2+^ (582 mg) > Pb^2+^ (652 mg kg^−1^) (Welp 1999). Although catalase belongs to the same class of oxidoreductases, it reacted extremely differently to the pressure caused by Sn^2+^, Co^2+^, and Mo^5^. Wyszkowska et al. (2009) also observed that this enzyme succumbed to the pressure of heavy metals, but not as negatively as dehydrogenases. Similar to the authors’ own research, arylsulfatase sensitivity to this group of xenobiotics was similar to acid phosphatase and alkaline phosphatase (Wyszkowska et al. 2010). It is supposed that low arylsulfatase activity values were not necessarily generated only by high doses of the metals. Knauff et al. (2003) claim that this could be caused by the release of H^+^ protons from barley roots, which reduces its activity, decreasing soil pH. Tabatabai (1977) stresses that Sn^2+^, Co^2+^, and Mo^6+^ all have an inhibitory effect on urease activity. The author proposed the following series of divalent ions as inhibitors of this enzyme: Ag^2+^ > Hg^2+^ > Cu^2+^ > Cd^2+^ > Zn^2+^ > Sn^2+^ > Mn^2+^. An attempt to explain the relationship between the time of soil residence of tested metals and decreasing values of microbiological indicators was made by Moreno et al. (2003). They claim that this may be related to the narrowing of the pool of substrates available to microorganisms.

**Fig. 4:**
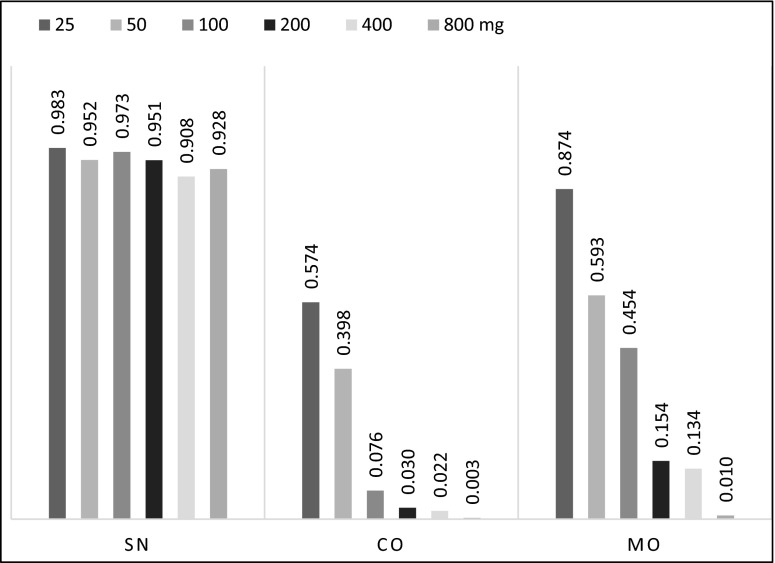
Index of resistance (RS) of spring barley depending on Sn^2+^, Co^2+^, and Mo^5+^ (mg kg^−1^ DM soil) pollution

The xenobiotics introduced into the soil, except for Sn^2+^, also contributed to a drastic growth inhibition of the cultivated plant (Fig. 4). Characteristic symptoms of the disturbances in the biological mechanisms of spring barley, as the effects of stress related to soil contamination with the discussed metals, were also root system deformation and leaf chlorosis. Only Sn^2+^, regardless of the metal dose, generated high spring barley RS values, oscillating between 0.908 and 0.983. Unquestionably, Co^2+^ proved most toxic which, even in the amount of 100 mg kg^−1^ soil DM, decreased this plant’s resistance sixfold compared to the objects with 25 mg Co^2+^ per kilogram soil DM, reducing its value almost to zero (0.076). An inhibitory effect was also observed for Mo^5+^, particularly in its higher doses, above 200 mg Mo^5+^ per kilogram soil DM. The response to Mo^5+^ toxicity was also observed by McGrath et al. (2010), who found that Mo^5+^ uptake by plants was closely correlated with soil pH and the antidote consisted of the competition between S^2+^ and Mo^5+^. In the presence of S^2+^ in soil, the absorption of this metal by a plant and, as a consequence, its inhibitory effect decreases. The results of own research correspond to those obtained by Li et al. (2009). Co^2+^ in an amount from 53.6 mg to 91 mg kg^−1^ soil DM, depending on soil type, caused 50 % spring barley growth inhibition. Mico et al. (2008) indicate that after the application of 45 mg Co^2+^ per kilogram soil DM the barley yield disappears. This metal mainly accumulates in plant roots (Lotfy and Mostafa 2014).

## Conclusions

The study results indicate that trace elements present in excess in soil are able to disturb its homeostasis. They verify both soil microbiological diversity and biochemical activity. After optimal amounts are exceeded, inhibition of its biological activity and, as a consequence, reduced plant yields can be expected.

Co^2+^ proved the most toxic. It generated not only very low enzyme activity and resistance values but also contributed to a drastic spring barley yield reduction. Mo^5+^, although it is an element participating in nitrogen and sulfur metabolism and is part of bacterial enzymes in increased amounts (similar to Co^2+^), became an important inhibitor of the activity of dehydrogenases and urease. Soil reaction to an excess of Sn^2+^ was also negative, but the scale of the problem was not as alarming. The grown plant reacted exceptionally positively to its increased soil doses. Spring barley resistance did not undergo significant changes.

To conclude, this experiment is an important link in a series of studies on the awareness of the quality of the environment in which we function. It reveals the existence of side effects to the contamination of soils with trace elements caused by the growing push towards an increased standard of living and consumerism.
